# The Function of Non-Coding RNAs in Lung Cancer Tumorigenesis

**DOI:** 10.3390/cancers11050605

**Published:** 2019-04-30

**Authors:** Cornelia Braicu, Alina-Andreea Zimta, Antonia Harangus, Ioana Iurca, Alexandru Irimie, Ovidiu Coza, Ioana Berindan-Neagoe

**Affiliations:** 1 Research Center for Functional Genomics, Biomedicine and Translational Medicine, “Iuliu Hatieganu” University of Medicine and Pharmacy, 23 Marinescu Street, 400337 Cluj-Napoca, Romania; braicucornelia@yahoo.com (C.B.); Harangus.antonia@umfcluj.ro (A.H.); ioanaiurca@yahoo.com (I.I.); 2 MEDFUTURE-Research Center for Advanced Medicine, “Iuliu Hatieganu” University of Medicine and Pharmacy, 23 Marinescu Street, 400337 Cluj-Napoca, Romania; andreea.zimta@umfcluj.ro; 3 “Leon Daniello” Pneumophtisiology Clinic, 6 Bogdan Petriceicu Hasdeu Street, 400332 Cluj-Napoca, Romania; 4 Tumor Biology Department, The Oncology Institute “Prof. Dr. Ion Chiricuta”, 34-36 Republicii Street, 400015 Cluj-Napoca, Romania; 5 11th Department of Oncological Surgery and Gynecological Oncology, “Iuliu Hatieganu” University of Medicine and Pharmacy, 34-36 Republicii Street, 400015 Cluj-Napoca, Romania; airimie@umfcluj.ro; 6 Department of Surgery, The Oncology Institute “Prof. Dr. Ion Chiricuta”, 34-36 Republicii Street, 400015 Cluj-Napoca, Romania; 7 “Iuliu Hatieganu” University of Medicine and Pharmacy, 4 Louis Pasteur Street, 400349 Cluj-Napoca, Romania; 8 Department of Radiotherapy with High Energies and Brachytherapy, The Oncology Institute “Prof. Dr. Ion Chiricuta”, 34-36 Republicii Street, 400015 Cluj-Napoca, Romania; 9 Department of Functional Genomics and Experimental Pathology, The Oncology Institute “Prof. Dr. Ion Chiricuta”, 34-36 Republicii Street, 400015 Cluj-Napoca, Romania

**Keywords:** lung cancer, circRNA, YRNA, snoRNA, T-UCR, piRNA, ncRNA

## Abstract

Lung cancer is the most prevalent and deadliest cancer worldwide. A significant part of lung cancer studies is dedicated to the expression alterations of non-coding RNAs. The non-coding RNAs are transcripts that cannot be translated into proteins. While the study of microRNAs and siRNAs in lung cancer received a lot of attention over the last decade, highly efficient therapeutic option or the diagnostic methods based on non-coding RNAs are still lacking. Because of this, it is of utmost importance to direct future research on lung cancer towards analyzing other RNA types for which the currently available data indicates that are essential at modulating lung tumorigenesis. Through our review of studies on this subject, we identify the following non-coding RNAs as tumor suppressors: ts-46, ts-47, ts-101, ts-53, ts-3676, ts-4521 (tRNA fragments), SNORD116-26, HBII-420, SNORD15A, SNORA42 (snoRNAs), piRNA-like-163, piR-35127, the piR-46545 (piRNAs), CHIAP2, LOC100420907, RPL13AP17 (pseudogenes), and uc.454 (T-UCR). We also found non-coding RNAs with tumor-promoting function: tRF-Leu-CAG, tRNA-Leu, tRNA-Val (tRNA fragments), circ-RAD23B, circRNA 100146, circPVT1, circFGFR3, circ_0004015, circPUM1, circFLI1, circABCB10, circHIPK3 (circRNAs), SNORA42, SNORA3, SNORD46, SNORA21, SNORD28, SNORA47, SNORD66, SNORA68, SNORA78 (snoRNAs), piR-65, piR-34871, piR-52200, piR651 (piRNAs), hY4 5’ fragments (YRNAs), FAM83A-AS1, WRAP53, NKX2-1-AS1 (NATs), DUXAP8, SFTA1P (pseudogene transcripts), uc.338, uc.339 (T-UCRs), and hTERC.

## 1. Introduction

Lung cancer is the malignancy with the highest incidence for both sexes at the global level, accounting for 11.6% of all cancer cases and it has the highest mortality rate, being responsible for 18.4% of all cancer-related deaths worldwide [1]. From a histological point of view, the dominant type of lung cancer is the non-small cell lung cancer (NSCLC, comprising 85% of total cancer cases), while small-cell lung cancer comprises 15% of lung tumors. NSCLC is composed of adenocarcinoma, squamous cell carcinoma and large-cell carcinoma [2]. 

The RNA world is far more complex than theorized at the beginning of modern-day molecular biology [3]. The protein-coding RNA originates from 3% of the genome. The remaining 97% constitutes the “dark matter” of molecular biology, having hidden information mainly responsible for phenotype regulation. With time, it was proven that the genome “dark matter” is transcribed into various RNA species [4], most of which do not encode for proteins. As follows, the RNA world is divided into two categories: the coding RNAs and the non-coding RNAs [5]. 

The protein-coding capacity of various RNA types still has an inconclusive status. While the messenger RNAs can encode for proteins, they were also proved to have intrinsic regulatory capacity. 

The transfer RNAs (tRNA), ribosomal RNA (rRNA) and spliceosomal RNAs were for a long time recognized as transcripts with non-coding capacity, but with well-established roles in the translation process. However, their status has changed over the last decade, with the discovery of tRNA and rRNA fragments, which can regulate gene expression. 

A significant portion of the genome “dark matter” along with the primary transcripts of mRNAs generates various RNA species. There are the short non-coding RNA (with a sequence less than 200 bp) and the long non-coding RNA (with a sequence more than 200 bp) [6]. The vast majority of these transcripts do not have protein-coding capacity, but they can regulate the molecular processes at the DNA-RNA-protein levels.

The short ncRNA category includes microRNA (miRNA), small interfering RNA (siRNA), piwi-interacting RNA (piRNA), small nucleolar RNA (snoRNA), YRNA, tiRNA, and tRF. The long non-coding RNAs are many times regarded as a single category of transcripts. However, the latest studies have shown that they are very heterogeneous and comprise a diversity of transcripts [7,8]. From this category, we have chosen to focus on: natural antisense transcript (NAT), transcribed ultraconserved region (T-UCR) and telomerase RNA component (TERC). 

The circular RNAs (circRNAs) and the pseudogene transcripts were for a long time categorized as non-coding RNAs [9,10], however the latest findings have shown that some circRNAs [11] and some pseudogene transcripts [12] can be translated into proteins. Because this applies only to a small fraction of these transcripts, they are nowadays regarded as non-coding RNAs with coding potential [11,12].

The study of non-coding RNAs and their role in modulating lung neoplasm development and progression has been extensively studied over the past few decades. In order to show the abundance of articles presented in this flied of studies, we used the Medical Subject Headings (MeSH) vocabulary used for indexing articles for PubMed. MeSH was used to select: “Lung Neoplasms” (MeSH) and “RNA, Untranslated” (MeSH) that recalled 5008 articles (date of access 25 March 2019), from 1967–2019. From the total of 5008 results, 2031 were not specific to our subject and thus were eliminated from our brief analysis. We looked at 2977 article which included original articles, reviews and meta-analysis. The reviews and meta-analysis may contain duplicates of original articles. Many other analyses based on these search items could be used, in order to demonstrate the debate in this field. 

Our MeSH (“Lung Neoplasms” (MeSH)) and “RNA, Untranslated” (MeSH)) literature research brought us to the following observations. Before 2000, researchers used ribozyme to modulate gene expression, however, with after 2000, with the discovery of non-coding RNAs, the modulation of gene expression was heavily influenced by the use of non-coding RNAs, with the research in this area knowing various trends. Between 2000 and 2010, the small interfering RNAs (siRNAs) were extensively studied as therapeutic options, due to their ability to target a single gene, however, nowadays, siRNAs are rarely proposed as therapy, but instead, they are used as a way to transitionally silence a gene in order to gain knowledge on its function. 

**Figure 1:**
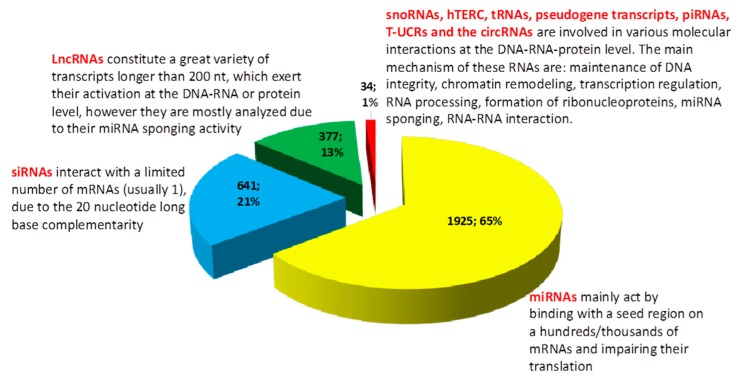
Graphic representation of the MeSH (PubMed comprehensive controlled vocabulary), results, by using the keywords “Lung Neoplasms” (MeSH)) and “RNA, Untranslated” (MeSH), from 5008 (date of access: 25.03.2019), we included in our brief analysis 2977 articles specific for our subject. More than 65% of studies (meaning 1925 articles from a total of 2977 investigated articles (based on MeSH vocabulary, “Lung Neoplasms” [Mesh] AND “RNA, Untranslated” [Mesh]), including original articles, reviews, and meta-analysis) analyzed the implication of various miRNAs in lung cancer (excluding studies which included the axis lncRNAs-miRNAs-mRNAs). At the same time, 21% of the current literature (meaning 641 articles from a total of 2977 investigated articles, including original articles, reviews and meta-analysis) have included siRNAs as a therapeutic option (from the total number of siRNAs studies we excluded the ones who had as their main subject the study of miRNAs). The “classic” lncRNAs (which includes a high heterogeneity of transcripts) are on the third place, comprising 13% of the total number of studies (377 out of 2977). CircRNAs are in the fourth place, but their number has continuously increased over the last year (20 studies from 2977). The lesser studied non-coding RNAs (snoRNAs, hTERC, tRNAs, piRNAs, pseudogene transcripts and T-UCRs) comprise only 0.47% of articles which analyze the role of non-coding RNAs in lung cancer; however, they are involved in various and multi-level molecular processes.

MicroRNAs are the most studied non-coding RNAs in lung cancer. Due to their ability to target multiple genes and their strong implication in cancer development, microRNAs have been frequently studied as potential biomarkers of lung neoplasm [13,14]. However, after the failure of the first human clinical trial with miRNA therapy and the challenges faced by the attempted standardization of miRNAs as biomarkers significantly impaired the translation of all of this knowledge into clinical practice and thus into significantly influencing the current prognostic of lung cancer patients. While the study of microRNAs in lung cancer is undeniably helpful, if we were to gain more knowledge on the role of other non-coding RNAs in lung neoplasm, the current status may significantly change in the future by a more complete picture of transcripts with tumorigenic role in lung cancer. A more graphic picture of these results and conclusions are depicted in Figure 1.

In our short review of the literature, we identified a problem. More than half of studies regarding non-coding RNAs in lung cancer looked at microRNAs, almost a quarter are using siRNAs in their studies and the long non-coding RNAs comprise only 13% of the total number of studies. In this context, approximately 1% of studies look at other RNA species, which might be equally important to microRNAs or siRNAs. More information and a general overlook over biogenesis and mechanism of action of these non-coding RNAs is found in Table 1.

We want to present in our review the biogenesis, molecular mechanism and the data on their involvement in lung cancer, with the purpose of encouraging future development of studies on these RNA species, which may lead to a more complete understanding of lung cancer and the formulation of better diagnostic and therapeutic options for the patients.

## 2. Transfer RNA 

Transfer RNA is non-coding RNA, known in the classical view of molecular biology as having the function of transporting the amino acids to the ribosome. However, this view was challenged by the latest discoveries of dysregulated expression of this RNA in various malignancies. Moreover, under intracellular conditions, the primary tRNAs are cleaved by angiogenin (ANG) and give rise to the tRNA-derived stress-induced RNAs (tiRNAs). The tiRNAs can remain as such, or they can be further processed by Dicer enzyme into tRNA-derived fragments (tRFs) [40]. 

Due to the high similarity between the two, some tRNAs are sometimes confused with microRNAs. For instance, with the help of Northern Blot, Pekarsky et al. demonstrated that miR-3676 and miR-4521 are actually, two tiRNAs, named ts-3676 and ts-4521. These tiRNAs are down-regulated in lung tumor tissue versus normal tissue in 17 analyzed cases and are mutated in six out of 300 lung cancer cases [41].

With the help of a custom microarray slide, it was found that ts-46 and ts-47 are down-regulated in lung cancer and that they act as tumor suppressors. The transfection of these transcripts in two lung cancer cell lines resulted in reduced proliferation rate and self-renewal capacity. Besides, ts-101 and ts-53 associate with PiwiL2, an essential protein involved in the silencing of transposons [42]. 

In lung tumor tissue, tRNAs-Leu and tRNAs-Val are overexpressed in 37% and 26% of samples. While the tRF-Leu-CAG is up-regulated in lung cancer tissue, serum, and lung cancer cell lines. Their nomenclature is derived from the anti-codon sequence and the amino acid to be transported. The increased expression of this tRF relates to disease progression, and it is involved in cell cycle progression and cell proliferation. tRF-Leu-CAG interacts with the AURKA protein [43] and can induce epithelial to mesenchymal transition (EMT) by regulating histone alteration via Wnt/β-catenin and PI3K/Akt pathway [44].

Specific degradation of some tRFs may have significant clinical application, as in the case of tRF-Leu-CAG, which suppresses lung cancer cell proliferation and cell cycle progression. There is still much to decipher related to biogenesis and role of tRF-Leu-CAG and other tRF transcripts [43].

## 3. Circular RNAs

CircRNAs are generated through back-splicing or lariat formation during alternative splicing. The cleaved part of the primary mRNA, containing either introns or exons or introns-exons, is circularized by various proteins belonging to the splicing machinery. CircRNAs has multiple mechanisms of action: it can interact with chromatin histones, bind to RNA polymerase, entrap the protein-coding exons from their originated mRNAs, sponge microRNAs, and entrap transcription factors in the cytoplasm and prevent gene transcription [37]. Most studies on circRNAs in lung cancer are focused on their miRNA sponging activity. 

In order to provide a better perspective over a broad range of molecular events with tumorigenic potential, an increasing number of studies evaluate the circRNA-miRNA-mRNA axis role in lung cancer. The circRAD23B is up-regulated in lung tumors versus normal adjacent tissue. This circRNA sponges miR-593-3p and miR-653-5p, and thus it restores the elevated expression of CCND2 mRNA and, respectively, TIAM1 mRNA [45]. The circRNA100146 is overexpressed in lung cancer tissue, and it sponges miR-361-3p and miR-615-5p. As a consequence of this, the miR-361-3p targets—NFAT5, COL1A1 and TRAF3- and miR-615-5p target—MEF2C- have increased expression in H460 cells [46]. circPVT1 leads to apoptosis resistance by sponging miR-497 a suppressor of the anti-apoptotic gene BCL-2 [47]. The circHIPK3, on the other hand, is a tumor-promoting circular RNA that has the ability of binding miR-124, miR-193, miR-379, and miR-654. The hsa_circ_0007385 is another oncogenic circRNA that acts as a ceRNA for miR-181. The hsa_circ_0013958 is up-regulated, and it leads to lung cancer proliferation and enhanced invasion capacity [48]. Circ_0000735 is overexpressed in tumor tissue versus normal tissue. This circRNA is positively associated with increased self-renewal capacity of malignant lung cells; thus it functions as an oncogene. At the molecular level, it sponges the tumor suppressor microRNAs, miR-1179 and miR-1182 [49]. A summary of the latest studies involving circRNA –miRNA-mRNA axis evaluation in lung cancer is found in Table 2.

On the other hand there are circRNAs that act as tumor suppressors due to their capacity to sponge oncogenic microRNAs. For example, the circ-ITCH is a tumor suppressor circular RNA that in lung cancer impairs disease progression by interacting with miR-7 and miR-214 [57].

Another mechanism of action of circRNAs in lung cancer is through interaction with transcription factors. For instance, the circNOL10 functions as a tumor suppressor in lung cancer, by impairing cell growth and decreasing in vivo tumor volume. The xenograft lung cancer tumors show a decreased expression of the anti-apoptotic gene BCL-2 and the pro-apoptotic gene, Casp3, and Bax. At the molecular level, circNOL10 prevents the ubiquitination of transcription factor sex comb on midleg-like 1 (SCML1) [58].

## 4. Small Nucleolar RNA (snoRNAs)

SnoRNAs are nucleotide sequences found in the introns of protein-coding or lncRNA coding genes. There are two mains kinds of snoRNAs: the C/D box snoRNAs and the H/ACA snoRNAs. Each class of snoRNA is associated with specific genes. The C/D box snoRNAs, abbreviated SNORD, contains the most significant number of members. The SNORDs contain A/C box (RUGAUGA, R = A/G), a C’ box (AGUAGU), a D box (AGUC) and a D’ box (CUGA). The C/D snoRNAs form a ribonucleoprotein which is bound with specific proteins such a Snu13p, Nop56p, Nop58p, and fibrillarin, in eukaryotic cells [59]. There are snoRNA for which no targets are found yet [28]. The H/ACA box snoRNAs is involved in the pre-rRNA pseudouridylation. The H region contains the following nucleotide sequence: ANANNA, where N = A/U/C/G. The SNORA has two hairpin structures, each with an internal loop; the pre-rRNA interacts with the pre-rRNA sequence in both hairpin structures, the conversion of uridine to pseudouridine is made in the internal loop, with the help of SNORA associated protein Cbf5p. The SNORA also form ribonucleoprotein with the Nop10pm Nhp2p and Gar1p proteins [60]. The snoRNAs are probably also involved in DNA replication and transcription. The p50 and p55 are two snoRNA associated proteins. The p55 was found to be associated with the TATA-binding proteins (TBP). The p55 and p50 also have helicase activity [61]. The human snoRNA is further processed into smaller miRNA-like fragments, which have approx 17–27 nt and originate in the majority of cases from the 5’ end of SNORD. The Dicer enzyme might be involved in the cleavage of snoRNA to smaller fragments, while the mechanism by which these snoRNAs are exported into the cytoplasm is still mostly unknown [62,63,64] The snoRNAs are also a source of piRNAs, as in the case of piR30840 [65]. Another unique function of these ncRNAs is a component of splicing machinery. Some C/D box snoRNAs use their methylase associated activity to methylate other RNA species or can lay the foundation of alternative splicing as in the case of the snoRNA, HBII-52 [66]. 

In lung cancer, the snoRNAs have profound implications. SNORD42 was proved to have an oncogenic role and to sustain lung cancer tumorigenesis [67]. A bioinformatic analysis based on TCGA data revealed that the U60, U63, U28, U51, U104, HBII-419, U59B, HBII-142, HBI-100, and U30 snoRNAs are up-regulated, while HBII-420 is down-regulated in lung cancer. Moreover, SNORD15A is significantly down-regulated in non-smoker tissue versus smoker tissue. The analysis also proved that in the pattern of snoRNAs differently expressed in normal tissue versus malignant tissue is more uniformly distributed in non-smokers versus smokers [68]. SNORD78 is up-regulated in lung cancer cells, and it leads to malignant cell proliferation, increased EMT and the consequent increase in invasion capacity [69]. Moreover, these transcripts are involved in maintaining the stem phenotype of the tumor-initiating cell. For instance, SNORD116-26 is underexpressed in tumor-initiating cells. Other snoRNAs, such as SNORA42 and SNORA3 are overexpressed in tumor-initiating cells. When SNORA42 is silenced in tumor-initiating cells, it results in decreased in vivo tumorigenesis of lung cancer cells. A lower expression of SNORA42 and SNORA3 in the human lung tumors increases the potential overall survival rate of lung cancer patients [70]. 

Through deep sequencing, it was established that the expression pattern of different snoRNA subtypes can predict the clinical progression of lung cancer. SNORD46 functions as an oncogene in lung cancer. The in vitro silencing on SNORD46 leads to decreased cell viability, inhibited invasion and migration capacity [71]. SnoRNAs have the capacity of predicting lung cancer progression from initial stages. Gao l. et al. showed that the overexpression of SNORA21, SNORD28, SNORA47, SNORD66, SNORA68, and SNORA78 leads to worse overall survival in lung cancer patients and that these snoRNAs can differentiate between lung tumors of stage I and normal tissue [72]. In the NSCLC patients, it was found SNORD33, SNORA42, SNORD66, and SNORD78 are overexpressed. SnoRNAs can be used as lung cancer biomarkers in combination with other miRNAs. Su et al. developed a panel of lung cancer biomarkers collected from sputum, which comprises three miRNAs (miR-21, miR-32, and miR-210) and two snoRNA (SNORD66, SNORD78) [73]. 

## 5. PIWI-Interacting RNAs 

The P-Element induced wimpy testes (PIWI) are proteins which can interact with the genome transposable elements (TE) and silence them. The interaction between PIWI proteins and the DNA strand is mediated by mutual interaction with the PIWI-interacting RNAs (piRNAs). These ncRNAs are originated from TE, mRNA or lncRNA. The primary form of piRNAs is a 200 kb single-stranded RNA. First, the primary transcript is cleaved at the 3′ end by Zucchini enzyme, is exported into the cytoplasm, and then it enters into the ping-pong amplification pathway [20,74,75].

Firstly, the antisense piRNA is loaded onto AGO3 and interacts with the sense piRNA through base pairing. The primary antisense transcript is cleaved at the 5′ end, then it is separated from the sense piRNA, and it is loaded on Aubergine (AUB) protein, where it is further cleaved from the 3’ end. After shortening, the complex of AUB-antisense piRNA interacts with the sense piRNA. The sense piRNA is first cleaved at the 5’end; then it is separated from AUB-antisense piRNA and loaded on AGO3. The AGO3-sense piRNA interacts again with a new antisense piRNA. The mature piRNA enters again into the nucleus [75,76,77].

The piR-34871 and piR-52200 are up-regulated, while the piR-35127 and the piR-46545 are down-regulated in lung cancer cells. RASSF1C has been demonstrated to be a tumor suppressor gene regulated by piRNAs, interfering with cell proliferation and apoptosis-related signaling pathways [78]. 

With the help of microarray analysis, it was demonstrated that piRNA-like-163 (piR-L-163) is one of the most down-regulated piRNAs in NSCLC. PiR-L-163 can bind directly to the phosphorylated form of ERM proteins (p-ERM) [79]. These proteins mediate the interaction between transmembrane proteins and cytoskeleton proteins [80]. The complex piR-L-163/p-ERM can bind to filamentous actin (F-actin) and ERM-binding phosphoprotein 50 (EBP50) thus causing cell migration [79]. The piR651 is overexpressed in lung cancer cell lines, and it is involved in apoptosis resistance. Exogenous silencing of piR651 leads to apoptosis activation through the overexpression of BAX and cleaved caspase 3, while the anti-apoptotic gene BCL-2 is down-regulated. The silencing of this gene also impairs cellular invasion and migration capacity of malignant lung cells [81]. 

Table 3 summarizes some studies, which evaluated the role of the lesser-known ncRNAs in lung cancer.

## 6. YRNA

YDNA is a set of four conserved DNA regions, which are transcribed by RNA polymerase III into YRNAs. They have around 100 nt: 112 nt (hY1), 101 nt (hY3), 93 nt (hY4) and 83 nt (hY5). The general structure of YRNA is: a loop domain, which is the least conserved, an upper stem domain, a lower stem domain and a polyuridine tail [25]. Depending on the type of RNA-nucleoprotein complex, YRNAs fulfill some functions inside the cell. The primary upper stem domain is involved in DNA replication, and the upper loop interacts with nucleolin, a protein associated with chromatin inside the nucleolus. The lower stem domain of YRNA interacts with Ro60 and detects misfolded RNA species. The polyuridine tail YRNA bind to La proteins and interacts with poly-U’tail of RNA III transcripts [94]. Other lesser-known YRNA-protein interactions are established with hnRNP I (PTBP1), RoBPI (PUF60), ZBP1 (IFGB2P1, IMP1), YBX1, YBX3, MOV10, Matrin-3, ELAVL1 (HuR), CPSF1, CPSF2, FIP1L1, SYMPK. These interactions are involved mainly in mRNA processing [95]. 

The extracellular vesicles play essential roles in the progression on various malignancies, lung cancer included [96,97]. The hY4 5′ fragments and YRNA fragments from hY4 pseudogenes were found in the extracellular vesicles isolated from the plasma of lung cancer patients. The hY4 was proved to stimulate the proliferation of lung cancer cells [82]. This is the only article published until now on the role of YRNAs in lung cancer, however, it indicates that these non-coding transcripts may fulfill essential functions in lung cancer progression. 

## 7. Natural Antisense Transcripts 

The natural antisense transcripts (NAT) are RNA sequences transcribed from the antisense strand of a coding strand. These transcripts were initially regarded as being non-functional, but recently it was found that these transcripts interact with their corresponding transcript from the sense strand [98]. NAT benefit from a low mutation status, similar to their strand counterparts. There are 797 conserved evolutionary NATs. They can influence the activity of the sense transcript from the same locus (cis-regulation) or of another transcript found at a distant site (trans-regulation). NAT has a different degree of overlapping with the sense transcript: full-length overlapping, tail-to-tail overlapping, head-to-head overlapping [99]. At the molecular level, NAT can have a concordant or a discordant regulation with the sense transcript, mediated by epigenetic regulation [100], entrapment of the transcription machinery, alteration of sense mRNA splicing and nuclear export [101], masking the miRNA binding sites on the sense mRNA or acting ceRNA for miRNAs targeting the sense mRNA [102]. 

This interaction can lead to the higher stability of the mRNA or translational repression of mRNA. For instance, the NKX2-1-AS1 is co-expressed with the tumor-promoting sense gene NKX2-1, and it offers greater stability to the NKX2-1 transcript. NKX2-1-AS1 is involved in lung cancer cell proliferation [84]. The WRAP53 is another NAT which contains fragments from the antisense strand of the tumor-suppressor p53, and it is up-regulated in lung cancer, leading to cell cycle arrest and proliferation [85]. However, the role of WRAP53 in lung cancer is dependent on p53 mutation status. The promoter methylation and the consequent WRAP53 down-regulation in WT p53 tumors is associated with a worse prognostic than in the case of tumors with normal expression of WRAP53, whereas in mutated p53 tumors, WRAP53 inhibition does not affect survival rate [103]. 

The NAT from FAM83A gene, FAM83A-AS1 is up-regulated in lung cancer tumor tissue versus healthy tissue. This NAT is positively correlated with the transcription and translation of the FAM83A gene thus increasing the intracellular level of FAM83A protein. This protein increases the phosphorilation of ERK1/2 [86].

## 8. Pseudogene Transcript

The pseudogenes are originated from coding-genes that have accumulated multiple mutations throughout evolution. The accumulation of mutation was associated with loss-of-function. However, this view is no longer valid; some research articles have concluded that alterations in the expression level of pseudogenes have pathological implications [104]. In comparison with somatic mutated genes, pseudogenes are conserved across species. The number of pseudogenes in the human genome is approximately 10,000–20,000. They can result from a single genome locus (unitary pseudogenes), from duplicated genes (duplicated pseudogenes), from the reverse transcription and retrotransposition of a mutate mRNAs. Approximately 20% of pseudogenes are transcribed, and they can inhibit the expression of parent genes, through multiple mechanisms: siRNA generation, acting as a ceRNA for miRNAs and the splicing machinery, antisense binding based on base complementarily to the corresponding mRNA [105].

**Figure 2:**
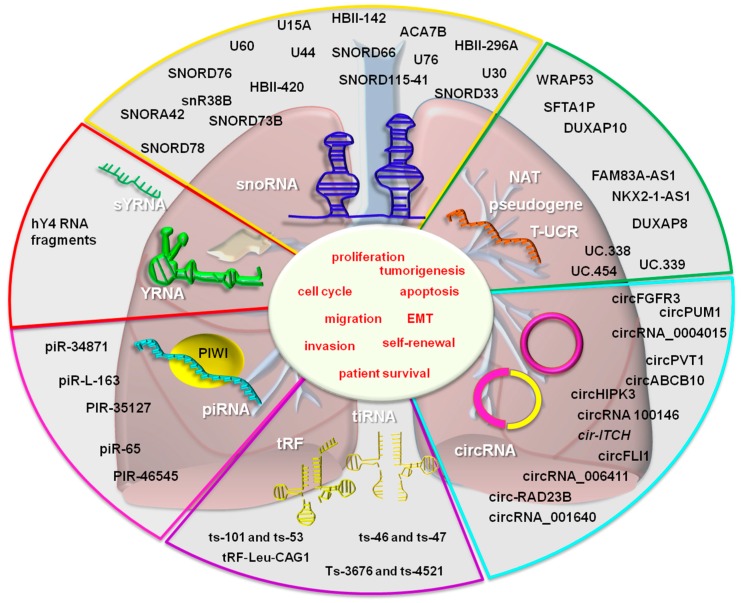
The lesser-known non-coding RNAs in lung cancer. The following non-coding RNAs function as tumor suppressors: ts-46, ts-47, ts-101, ts-53, ts-3676, ts-4521 (tRNA fragments), SNORD116-26, HBII-420, SNORD15A, SNORA42 (snoRNAs), piRNA-like-163, piR-35127, the piR-46545 (piRNA), CHIAP2, LOC100420907, RPL13AP17 (pseudogenes), and Uc.454 (T-UCR). The oncogenic non-coding RNAs from the above mentioned categories are: tRF-Leu-CAG, tiRNAs-Leu, tiRNAs-Val (tRNA fragments), circ-RAD23B, circRNA 100146, circPVT1, circFGFR3, circ_0004015, circPUM1, circFLI1, circABCB10, circHIPK3 (circRNAs), SNORA42, SNORA3, SNORD46, SNORA21, SNORD28, SNORA47, SNORD66, SNORA68, SNORA78 (snoRNAs), piR-65, piR-34871, piR-52200, piR651 (piRNAs), hY4 5′ fragments (YRNA), FAM83A-AS1, WRAP53, NKX2-1-AS1 (NAT), DUXAP8, SFTA1P (pseudogene transcript) and, Uc.454 (T-UCR), her.

A summary of lesser-known non-coding RNAs in lung cancer is found in Figure 2.

Because of their high similarity to the rapidly evolving organism, cancer cells can accumulate pseudogene more rapidly in comparison with a normal cell, through a process called somatic retrotransposition [106]. The transcribed pseudogene SFTA1P is up-regulated in lung cancer. This increased expression lowers the patient’s survival. During in vitro experiments, it was proved that the inhibition of SFTA1P causes decreased proliferation, migration, and invasion of lung cancer cells [88]. 

The transcribed pseudogene called DUXAP8 is upregulated in lung cancer tissues, being related with increased cells proliferation and invasion capacity through epigenetic silencing of EGR1 and RHOB genes [89]. Up-regulation of DUXAP10 leads to a worse survival rate in lung cancer patients. DUXAP10 has an oncogenic role by interacting with Histone demethylase Lysine-specific demethylase1 (LSD1) and repression of Large tumor suppressor 2 (LATS2) and Ras-related associated with diabetes (RRAD) transcription [90]. 

CHIAP2, LOC100420907, and RPL13AP17 pseudogenes are down-regulated in lung cancer and are positively associated with overall patient’s survival rate. CHIAP2 experimentally induced overexpression correlates with both miR-873-3p and miR-3614-5p inhibition. miR-3614-5p functions as a tumor suppressor in lung cancer, while miR-873 is an oncogene in lung cancer [107]. The cold shock domain protein A intronless pseudogene (CSDAP1) is overexpressed in lung cancer tissue, and it is positively correlated with disease progression and patients overall survival rate [108].

## 9. Transcribed Ultraconserved Region (T-UCR)

The ultraconserved regions of the DNA are genome loci which bear a high degree of similarity across species [109], more precisely, they have 100% similarity at orthologous regions across distant related mammalian species (such as human, mouse, rat). In the human genome, there are 481 UCRs which generate long non-coding RNAs, named transcribed ultra conserved regions (T-UCRs). They are generally silenced in cancer tissue due to hypermethylation of CpG island and miRNA interaction. Mutations in UCRs have many downstream effects in cancer pathogenesis thus proving their significant role in maintaining cell homeostasis [109].

Uc.338 is overexpressed in lung cancer. The siRNA mediated silencing of uc.338 lead to inhibition of cell proliferation, cell cycle progression, invasion and migration in vitro. These were correlated with a decreased expression of cells-else related genes: cyclin B1 and CDC25C. At the same time, the expression of mesenchymal-associated genes: Snail, VIM and N-CAD were inhibited and the expression of an epithelial-associated gene, E-CAD was increased [91]. The expression of uc.339 is up-regulated in NSCLC. TP53 controls its transcription through the interaction with a consensus sequence found 2963 bp upstream of uc.339 gene. Uc.339 acts as a ceRNA for miR-339, miR-663b, and miR-95; however, these microRNAs do not affect uc.339 expression. By entrapping these miRNAs, their target gene cyclin E2 has a higher expression level thus leading to increased cell cycle progression and migration capacity in lung cancer cells [92].

Uc.454 is a T-UCR down-regulated in lung cancer tissue and lung cancer cell lines, being associated with the clinical stage of the disease. In vitro transfection of lung cancer cells with uc.454 mimic decreased the self-renewal capacity of cells and increased apoptosis, while in vivo caused the formation of smaller tumor compared to the control. It was found that the observed effects of uc.454 are mediated by its interaction with the heat shock protein, HSPA12B [93]. 

## 10. Telomerase RNA Component

The telomerase RNA component (TERC) is associated with the telomerase enzyme. This enzyme maintains chromosome length thus providing replicative immortality to malignant cells [110] and surpassing the Hayflick limit. Due to the high importance of maintaining TERC integrity throughout multiple cell divisions, TERC contains a highly conserved region termed Stem Terminus Element (STE) [111]. 

By analyzing three lung cancer cell lines, a study demonstrated the binding of CTCF TF to one of the enhancer regions of TERT gene is responsible for TERT up-regulation [112]. Some genetic variants of TERC (rs10936599) or TERT (rs10069690, rs2242652 or rs2853677) are related with an increased lung cancer risk [113]. 

In lung cancer, the enhancer-promoter interaction of TERT gene leads to its up-regulation. The Zinc-finger protein Snail1 prevents this interaction, but when Snail1 has an inherited mutation in its binding site, it leads to lung cancer initiation, in vitro [114]. However, a study found that hTERC has the same level of expression in lung tumor tissue and healthy tissue, and it is not correlated to telomerase activity [115].

## 11. Conclusions and Perspectives

The discovery of non-coding RNAs and their role in cancer initiation, progression, and therapeutic response has revolutionized the current research directions and clinical management of lung cancer. However, the data is still incomplete due to the fact that most of these studies focus on microRNAs and siRNAs as opposed to other types of non-coding RNAs.

As follows, we reviewed the currently available literature on tRNA fragments, snoRNAs, circRNAs, NATs, pseudogenes, T-UCRs, piRNAs, YRNA fragments and YRNAs. We found that these RNAs are involved in multi-level modulation of malignant cell behavior: epigenetic silencing/stimulation, processing of mRNAs, acting as ceRNAs for miRNAs, stabilizing the mRNA, interacting with proteins and determining the cellular localization of proteins.

The following non-coding RNAs function as tumor suppressors: ts-46, ts-47, ts-101, ts-53, ts-3676, ts-4521 (tRNA fragments), SNORD116-26, HBII-420, SNORD15A, SNORA42 (snoRNAs), piRNA-like-163, piR-35127, the piR-46545 (piRNAs), CHIAP2, LOC100420907, RPL13AP17 (pseudogenes), and uc.454 (T-UCR). The non-coding RNA with oncogenic role in lung tumorigenesis are: tRF-Leu-CAG, tRNA-Leu, tRNA-Val (tRNA fragments), circ-RAD23B, circRNA 100146, circPVT1, circFGFR3, circ_0004015, circPUM1¸ circFLI1, circABCB10, circHIPK3 (circRNAs), SNORA42, SNORA3, SNORD46, SNORA21, SNORD28, SNORA47, SNORD66, SNORA68, SNORA78 (snoRNAs), piR-65, piR-34871, piR-52200, piR651 (piRNAs), hY4 5′ fragments (YRNA), FAM83A-AS1, WRAP53, NKX2-1-AS1 (NATs), DUXAP8, SFTA1P (pseudogene transcripts), uc.338, uc.339 (T-UCRs), and hTERC.

Although the role of miRNAs and siRNAs in lung cancer has been extensively studied and have well-defined roles, there are many RNAs which also affect cancer cell behavior but have been for a long time overlooked by the scientific community. As follows, the RNA world is far more complex and the clinical setting of lung cancer progression should be much improved if more research would be dedicated to the study of lesser-known non-coding RNAs and their involvement in lung malignancy.

## Figures and Tables

**Table 1:** The main types of non-coding RNAs and their related characteristics.

Type of RNAs	Length	Region of the DNA	Localization	Interaction	Molecular Role	Ref.
Short ncRNAs						
miRNA	22–24 nt	Intergenic regions and introns of protein-coding genes.	Nucleus, Cytoplasm	mRNA, circRNA, NAT, pseudogene transcript, T-UCR	Translation suppression	[15,16]
siRNA	20–30 nt	Pseudogenes, intergenic repetitive sequence, endo-siRNA gene clusters	Cytoplasm	mRNA	Translation repression	[17,18]
piRNA	24–31 nt	Flamenco locus, containing fragmented transposons	Cytoplasm	mRNA	Translation repression, modulation of transposons	[19,20]
tRF	20 nt	tRNA coding transcripts	Nucleus	mRNA, transportable elements	Cell proliferation, translation repression, target transportable element	[21,22]
tiRNA	30–40 nt	tRNA coding transcripts	Cytoplasm	mRNA, tRF	Translation repression, signaling molecule	[23]
YRNA	84–112 nt	yDNA	Nucleus	DNA, primary RNA transcripts	Misfolded RNA degradation, DNA replication	[24,25,26]
snoRNA	60–200 nt C/D snoRNAs, 120–250 nt for H/ACA snoRNAs	introns, promoter region of Pol II	Nucleus, Cytoplasm	mRNA, DNA, primary transcripts	Transcription regulation by binding to TATA box, rRNA processing, splicing, miRNA-like functions, chromatin remodeling, DNA replication, generation of piRNA/miRNA	[27,28]
Long non-coding RNA						
TERC	451 nt	promoter region of pol II	Nucleus	DNA, telomerase	Telomere length maintenance	[29,30]
NAT	>200 nt Depending on the antisense gene length	antisense strand of protein-coding transcript	Nucleus, Cytoplasm	DNA, miRNA, mRNA	Inhibition of the mRNA, epigenetic gene silencing, masking miRNA binding site on the mRNA, entrapment of splicing machinery	[31,32,33]
T-UCR	>200 nt	ultraconserved regions of the DNA	Cytoplasm	miRNA	miRNA sponge	[34]
Long non-coding RNAs with coding potential						
circRNA	200–800 nt	circular RNA	Nucleus, Cytoplasm	miRNA, mRNA, rRNA	Epigenetic silencing of genes, miRNA sponge, Translation repression, protein-coding function, protein scaffold	[35,36,37]
Pseudogene transcripts	>200 nt depending on the pseudogene length	Pseudotranscripts/pseudogene transcripts	Nucleus, Cytoplasm	miRNA, siRNA,	Translation repression, miRNA sponge, generation of miRNA/siRNA	[38,39]

**Table 2:** Examples of oncogenic circRNAs which act as miRNA sponges in lung cancer.

Name	Targeted miRNA	Indirect Effect Over mRNA	Biological Effect	Reference
circ-RAD23B	miR-593-3p	CCND2	Promotes invasion	[45]
	miR-653-5p	TIAM1		
circRNA 100146	miR-361-3p	NFAT5, COL1A1, TRAF3	Promotes invasion and cell proliferation	[46]
	miR-615-5p	MEF2C		
circPVT1	miR-497	BCL-2	Promotes apoptosis, impairs proliferation	[47]
	miR-125b	E2F1	Stimulated in vivo tumorigenesis	[50]
circFGFR3	miR-22-3p	Gal-1, p-AKT, and p-ERK1/2	Promotes invasion	[51]
circ_0004015	miR-1183	PDPK1	Decreases survival rate, promotes viability, proliferation, invasion and maybe gefitinib resistance	[52]
circPUM1	miR-326	CCND1 and BCL-2	Promotes proliferation, invasion and migration	[53]
circFLI1	miR-584-3p	ROCK1	Promotes metastasis	[54]
circABCB10	miR-1252	FOXR2	Promotes proliferation and migration	[55]
circHIPK3	miR-124	SphK1, STAT3 and CDK4	Promotes proliferation and impairs apoptosis	[56]

**Table 3:** Some example of altered ncRNAs involved in lung cancer.

Type of RNAs	Transcript	Up/Down	Biological Role	Reference
**piRNA**	piR-34871, piR-52200	UP	It stimulates cell proliferation and apoptosis via RASSF1C gene	[78]
	piR-35127, piR-46545	DOWN	There was no experimental modulation of its expression.	[78]
	piR-L-163	DOWN	It binds to p-ERM proteins that interact with transmembrane and cytoskeleton proteins thus impairing cell migration, cell cycle progression;	[79]
**tRNA fragments**	ts-46, ts-47, ts-101 and ts-53	DOWN	It impairs cell proliferation	[42]
	tRF-Leu-CAG1 tRF-Leu-CAG2	UP	It stimulates cell cycle progression and cell proliferation; by targeting AURKA protein	[43]
**YRNA**	hY4 RNA	UP	It was found in the extracellular vesicle from lung tumor cells, it stimulates cell proliferation	[82]
**SNORD**	SNORA42	UP	It maintains the tumor initiating phenotype of cancer cells	[70]
	U60, U63, U28, U51, U104, HBII-419, U59B, HBII-142, HBI-100, U30	UP	There was no experimental modulation of its expression.	[68]
	HBII-420	DOWN		
	SNORD78	UP	It increases the in vivo tumorigenesis, and in vitro lung malignant cell proliferation, cell cycle progression, invasion, self-renewal capacity, and maintenance of cell steamness	[69]
	SNORA47, SNORA68, SNORA78, SNORA21, SNORD28 SNORD66	UP	There was no experimental modulation of its expression.	[72]
	SNORD33, SNORD66 SNORD76	UP	There was no experimental modulation of its expression.	[83]
**NAT**	NKX2-1-AS1	UP	It increases lung malignant cell proliferation rate	[84]
	WRAP53		It increases lung malignant cell proliferation rate	[85]
	FAM83A		It causes in vivo pronounced tumor progression	[86]
	AFAP1-AS1		It causes increased invasion and metastasis capacity of lung malignant cells	[87]
**Pseudogene transcripts**	SFTA1P	DOWN	It impairs cell migration and invasion	[88]
	DUXAP8	UP	It increases malignant cell survival and proliferation, it stimulates in vivo tumorigenesis	[89]
	DUXAP10		It increases malignant cell survival, proliferation, and migration, it stimulates in vivo tumorigenesis	[90]
**T-UCR**	Uc.338	UP	It increases malignant cell cycle progression, invasion, and migration	[91]
	Uc.339	UP	It increases malignant cell cycle progression, and migration	[92]
	Uc.454	DOWN	It decreases malignant cell cycle progression, invasion, and migration	[93]

## Abbreviations

NSCLC	non-small cell lung cancer
ncRNA	non-coding RNA
nt	nucleotide
bp	base pairs
TF	transcription factor
tRNA	transfer RNA
rRNA	ribosomal RNA
miRNA/miR	microRNA
siRNA	small interfering RNA
piRNA	piwi-interacting RNA
piR	piwi-interacting RNA
snoRNA	small nucleolar RNA
circRNA	circular RNA
tRF	tRNA-derived fragments
half-tRNA	tiRNA
ceRNA	competing endogenous RNA
NAT	natural antisense transcripts
T-UCR	transcribed ultraconserved region
dsRNA	double-stranded RNA
shRNA	short hairpin RNA
TERC	telomerase associated RNA
NAT	natural antisense transcripts
p-ERM	phosphorylated ERM proteins
NXN	nucleoredoxin
AHRR	Aryl-Hydrocarbon Receptor Repressor
CDC42EP3	CDC42 Effector Protein 3
EDC3	Enhancer of MRNA Decapping 3
HIPK2	Homeodomain Interacting Protein Kinase 2
CXCL5	C-X-C Motif Chemokine Ligand 5
SEC62	SEC62 Homolog: Preprotein Translocation Factor
ZNF703	Zinc Finger Protein 703
CTNNB	Catenin Beta 1
BRD	bromodomain
BET	extra-terminal
PTEN	Phosphatase and Tensin Homolog
TNC	Tenascin C
TERC	telomerase RNA component
BAX	BCL2 Associated X: Apoptosis Regulator
CCND2	Cyclin D
TIAM1	T-lymphoma invasion and metastasis-inducing protein 1
NFAT5	Nuclear factor of activated T-cells 5
CO1A1	Collagen alpha-1(I) chain
TRAF3	TNF Receptor Associated Factor 3
MEF2C	Myocyte Enhancer Factor 2C
RPCK1	Rho-associated protein kinase 1
FOXR2	Forkhead Box R2
SphK1	Sphingosine kinase 1
STAT3	Signal transducer and activator of transcription 3
CDK4	Cyclin-dependent kinase 4
NKX2-1	NK2 homeobox 1
DUXAP8	Double Homeobox A Pseudogene 8
EGR1	Early growth response protein 1
RHOB	Rho-related GTP-binding protein RhoB
LSD1	Histone demethylase Lysine specific demethylase1
LATS2	Large tumour suppressor 2
RRAD	Ras-related associated with diabetes
CHIAP2	Chitinase: Acidic Pseudogene 2
RPL13AP17	Ribosomal Protein L13a Pseudogene 17
p-ERM	phosphorylated ERM proteins
EMT	epithelial to mesenchymal transition
Ect2	Epithelial Cell Transforming Sequence 2
EV	extracellular vesicles

## References

[1] Bray F., Ferlay J., Soerjomataram I., Siegel R.L., Torre L.A., Jemal A. (2018). Global cancer statistics 2018: GLOBOCAN estimates of incidence and mortality worldwide for 36 cancers in 185 countries. CA Cancer J. Clin..

[2] Alberg A.J., Brock M.V., Samet J.M. (2005). Epidemiology of lung cancer: Looking to the future. J. Clin. Oncol. Off. J. Am. Soc. Clin. Oncol..

[3] Crick F. (1970). Central dogma of molecular biology. Nature.

[4] Ling H., Girnita L., Buda O., Calin G.A. (2017). Non-coding RNAs: The cancer genome dark matter that matters!. Clin. Chem. Lab. Med..

[5] Hubé F., Francastel C. (2018). Coding and Non-coding RNAs, the Frontier Has Never Been So Blurred. Front. Genet..

[6] Brosnan C.A., Voinnet O. (2009). The long and the short of noncoding RNAs. Curr. Opin. Cell Biol..

[7] Wu H., Yang L., Chen L.L. (2017). The Diversity of Long Noncoding RNAs and Their Generation. Trends Genet.

[8] Uszczynska-Ratajczak B., Lagarde J., Frankish A., Guigo R., Johnson R. (2018). Towards a complete map of the human long non-coding RNA transcriptome. Nat. Rev. Genet..

[9] Chen B., Huang S. (2018). Circular RNA: An emerging non-coding RNA as a regulator and biomarker in cancer. Cancer Lett..

[10] Groen J.N., Capraro D., Morris K.V. (2014). The emerging role of pseudogene expressed non-coding RNAs in cellular functions. Int. J. Biochem. Cell Biol..

[11] Das A., Gorospe M., Panda A.C. (2018). The coding potential of circRNAs. Aging.

[12] Xu J., Zhang J. (2016). Are Human Translated Pseudogenes Functional?. Mol. Biol. Evol..

[13] Sonea L., Buse M., Gulei D., Onaciu A., Simon I., Braicu C., Berindan-Neagoe I. (2018). Decoding the Emerging Patterns Exhibited in Non-coding RNAs Characteristic of Lung Cancer with Regard to their Clinical Significance. Current Genom..

[14] Redis R.S., Berindan-Neagoe I., Pop V.I., Calin G.A. (2012). Non-coding RNAs as theranostics in human cancers. J. Cellular Biochem..

[15] Starega-Roslan J., Krol J., Koscianska E., Kozlowski P., Szlachcic W.J., Sobczak K., Krzyzosiak W.J. (2011). Structural basis of microRNA length variety. Nucleic Acids Res..

[16] Macfarlane L.A., Murphy P.R. (2010). MicroRNA: Biogenesis, Function and Role in Cancer. Curr. Genom..

[17] Dana H., Chalbatani G.M., Mahmoodzadeh H., Karimloo R., Rezaiean O., Moradzadeh A., Mehmandoost N., Moazzen F., Mazraeh A., Marmari V. (2017). Molecular Mechanisms and Biological Functions of siRNA. Int. J. Biomed. Sci. Ijbs.

[18] Kim V.N., Han J., Siomi M.C. (2009). Biogenesis of small RNAs in animals. Nat. Rev. Mol. Cell Biol..

[19] Iwasaki Y.W., Siomi M.C., Siomi H. (2015). PIWI-Interacting RNA: Its Biogenesis and Functions. Annu. Rev. Biochem..

[20] Ozata D.M., Gainetdinov I., Zoch A., O’Carroll D., Zamore P.D. (2019). PIWI-interacting RNAs: Small RNAs with big functions. Nat. Rev. Genet..

[21] Lee Y.S., Shibata Y., Malhotra A., Dutta A. (2009). A novel class of small RNAs: tRNA-derived RNA fragments (tRFs). Genes Dev..

[22] Martinez G., Choudury S.G., Slotkin R.K. (2017). tRNA-derived small RNAs target transposable element transcripts. Nucleic Acids Res..

[23] Saikia M., Hatzoglou M. (2015). The Many Virtues of tRNA-derived Stress-induced RNAs (tiRNAs): Discovering Novel Mechanisms of Stress Response and Effect on Human Health. J. Biol. Chem..

[24] Dhahbi J.M., Spindler S.R., Atamna H., Boffelli D., Mote P., Martin D.I. (2013). 5’-YRNA fragments derived by processing of transcripts from specific YRNA genes and pseudogenes are abundant in human serum and plasma. Physiol. Genom..

[25] Kowalski M.P., Krude T. (2015). Functional roles of non-coding Y RNAs. Int. J. Biochem. Cell Biol..

[26] Stein A.J., Fuchs G., Fu C., Wolin S.L., Reinisch K.M. (2005). Structural insights into RNA quality control: The Ro autoantigen binds misfolded RNAs via its central cavity. Cell.

[27] Scott M.S., Ono M. (2011). From snoRNA to miRNA: Dual function regulatory non-coding RNAs. Biochimie.

[28] Dupuis-Sandoval F., Poirier M., Scott M.S. (2015). The emerging landscape of small nucleolar RNAs in cell biology. Wiley Interdiscip. Rev. RNA.

[29] Rubtsova M.P., Vasilkova D.P., Naraykina Y.V., Dontsova O.A. (2016). Peculiarities of Yeasts and Human Telomerase RNAs Processing. Acta Nat..

[30] Tseng C.K., Wang H.F., Burns A.M., Schroeder M.R., Gaspari M., Baumann P. (2015). Human Telomerase RNA Processing and Quality Control. Cell Rep..

[31] Rao X., Huang D., Sui X., Liu G., Song X., Xie J., Huang D. (2014). Overexpression of WRAP53 is associated with development and progression of esophageal squamous cell carcinoma. PLoS ONE.

[32] Piatek M.J., Henderson V., Zynad H.S., Werner A. (2016). Natural antisense transcription from a comparative perspective. Genomics.

[33] Wight M., Werner A. (2013). The functions of natural antisense transcripts. Essays Biochem..

[34] Terracciano D., Terreri S., de Nigris F., Costa V., Calin G.A., Cimmino A. (2017). The role of a new class of long noncoding RNAs transcribed from ultraconserved regions in cancer. Biochim. Et Biophys. Acta. Rev. Cancer.

[35] Memczak S., Papavasileiou P., Peters O., Rajewsky N. (2015). Identification and Characterization of Circular RNAs As a New Class of Putative Biomarkers in Human Blood. PLoS ONE.

[36] Zhang Y., Liang W., Zhang P., Chen J., Qian H., Zhang X., Xu W. (2017). Circular RNAs: Emerging cancer biomarkers and targets. J. Exp. Clin. Cancer Res. Cr.

[37] Braicu C., Zimta A.A., Gulei D., Olariu A., Berindan-Neagoe I. (2019). Comprehensive analysis of circular RNAs in pathological states: Biogenesis, cellular regulation, and therapeutic relevance. Cell. Mol. Life Sci. Cmls.

[38] Jingsi T., Mingyao Y., Ying L. (2015). Functional roles of pseudogenes in cancers. Yi Chuan Hered..

[39] Xiao-Jie L., Ai-Mei G., Li-Juan J., Jiang X. (2015). Pseudogene in cancer: Real functions and promising signature. J. Med. Genet..

[40] Huang S.Q., Sun B., Xiong Z.P., Shu Y., Zhou H.H., Zhang W., Xiong J., Li Q. (2018). The dysregulation of tRNAs and tRNA derivatives in cancer. J. Exp. Clin. Cancer Res. Cr.

[41] Pekarsky Y., Balatti V., Palamarchuk A., Rizzotto L., Veneziano D., Nigita G., Rassenti L.Z., Pass H.I., Kipps T.J., Liu C.G. (2016). Dysregulation of a family of short noncoding RNAs, tsRNAs, in human cancer. Proc. Natl. Acad. Sci. USA.

[42] Balatti V., Nigita G., Veneziano D., Drusco A., Stein G.S., Messier T.L., Farina N.H., Lian J.B., Tomasello L., Liu C.G. (2017). tsRNA signatures in cancer. Proc. Natl. Acad. Sci. USA.

[43] Shao Y., Sun Q., Liu X., Wang P., Wu R., Ma Z. (2017). tRF-Leu-CAG promotes cell proliferation and cell cycle in non-small cell lung cancer. Chem. Biol. Drug Des..

[44] Liu X., Li Z., Song Y., Wang R., Han L., Wang Q., Jiang K., Kang C., Zhang Q. (2016). AURKA induces EMT by regulating histone modification through Wnt/beta-catenin and PI3K/Akt signaling pathway in gastric cancer. Oncotarget.

[45] Han W., Wang L., Zhang L., Wang Y., Li Y. (2019). Circular RNA circ-RAD23B promotes cell growth and invasion by miR-593-3p/CCND2 and miR-653-5p/TIAM1 pathways in non-small cell lung cancer. Biochem. Biophys. Res. Commun..

[46] Chen L., Nan A., Zhang N., Jia Y., Li X., Ling Y., Dai J., Zhang S., Yang Q., Yi Y. (2019). Circular RNA 100146 functions as an oncogene through direct binding to miR-361-3p and miR-615-5p in non-small cell lung cancer. Mol. Cancer.

[47] Qin S., Zhao Y., Lim G., Lin H., Zhang X., Zhang X. (2019). Circular RNA PVT1 acts as a competing endogenous RNA for miR-497 in promoting non-small cell lung cancer progression. Biomed. Pharmacother. Biomed. Pharmacother..

[48] Zhu X., Wang X., Wei S., Chen Y., Chen Y., Fan X., Han S., Wu G. (2017). hsa_circ_0013958: A circular RNA and potential novel biomarker for lung adenocarcinoma. Febs J..

[49] Li W., Jiang W., Liu T., Lv J., Guan J. (2019). Enhanced expression of circ_0000735 forecasts clinical severity in NSCLC and promotes cell progression via sponging miR-1179 and miR-1182. Biochem. Biophys. Res. Commun..

[50] Li X., Zhang Z., Jiang H., Li Q., Wang R., Pan H., Niu Y., Liu F., Gu H., Fan X. (2018). Circular RNA circPVT1 Promotes Proliferation and Invasion Through Sponging miR-125b and Activating E2F2 Signaling in Non-Small Cell Lung Cancer. Cell. Physiol. Biochem..

[51] Qiu B.Q., Zhang P.F., Xiong D., Xu J.J., Long X., Zhu S.Q., Ye X.D., Wu Y., Pei X., Zhang X.M. (2018). CircRNA fibroblast growth factor receptor 3 promotes tumor progression in non-small cell lung cancer by regulating Galectin-1-AKT/ERK1/2 signaling. J. Cell. Physiol..

[52] Zhou Y., Zheng X., Xu B., Chen L., Wang Q., Deng H., Jiang J. (2019). Circular RNA hsa_circ_0004015 regulates the proliferation, invasion, and TKI drug resistance of non-small cell lung cancer by miR-1183/PDPK1 signaling pathway. Biochem. Biophys. Res. Commun..

[53] Chen J., Xu S., Chen S., Zong Z., Han X., Zhao Y., Shang H. (2019). CircPUM1 promotes the malignant behavior of lung adenocarcinoma by regulating miR-326. Biochem. Biophys. Res. Commun..

[54] Li L., Li W., Chen N., Zhao H., Xu G., Zhao Y., Pan X., Zhang X., Zhou L., Yu D. (2019). FLI1 Exonic Circular RNAs as a Novel Oncogenic Driver to Promote Tumor Metastasis in Small Cell Lung Cancer. Clin. Cancer Res. Off. J. Am. Assoc. Cancer Res..

[55] Tian X., Zhang L., Jiao Y., Chen J., Shan Y., Yang W. (2019). CircABCB10 promotes nonsmall cell lung cancer cell proliferation and migration by regulating the miR-1252/FOXR2 axis. J. Cell. Biochem..

[56] Yu H., Chen Y., Jiang P. (2018). Circular RNA HIPK3 exerts oncogenic properties through suppression of miR-124 in lung cancer. Biochem. Biophys. Res. Commun..

[57] Wan L., Zhang L., Fan K., Cheng Z.X., Sun Q.C., Wang J.J. (2016). Circular RNA-ITCH Suppresses Lung Cancer Proliferation via Inhibiting the Wnt/beta-Catenin Pathway. Biomed Res. Int..

[58] Nan A., Chen L., Zhang N., Jia Y., Li X., Zhou H., Ling Y., Wang Z., Yang C., Liu S. (2019). Circular RNA circNOL10 Inhibits Lung Cancer Development by Promoting SCLM1-Mediated Transcriptional Regulation of the Humanin Polypeptide Family. Adv. Sci. (Weinh. Baden-Wurtt. Ger.).

[59] Russell A.G., Watanabe Y., Charette J.M., Gray M.W. (2005). Unusual features of fibrillarin cDNA and gene structure in Euglena gracilis: Evolutionary conservation of core proteins and structural predictions for methylation-guide box C/D snoRNPs throughout the domain Eucarya. Nucleic Acids Res..

[60] Khanna M., Wu H., Johansson C., Caizergues-Ferrer M., Feigon J. (2006). Structural study of the H/ACA snoRNP components Nop10p and the 3’ hairpin of U65 snoRNA. RNA.

[61] Newman D.R., Kuhn J.F., Shanab G.M., Maxwell E.S. (2000). Box C/D snoRNA-associated proteins: Two pairs of evolutionarily ancient proteins and possible links to replication and transcription. RNA.

[62] Taft R.J., Glazov E.A., Lassmann T., Hayashizaki Y., Carninci P., Mattick J.S. (2009). Small RNAs derived from snoRNAs. RNA.

[63] Bai B., Yegnasubramanian S., Wheelan S.J., Laiho M. (2014). RNA-Seq of the nucleolus reveals abundant SNORD44-derived small RNAs. PLoS ONE.

[64] Martens-Uzunova E.S., Olvedy M., Jenster G. (2013). Beyond microRNA--novel RNAs derived from small non-coding RNA and their implication in cancer. Cancer Lett..

[65] Zhong F., Zhou N., Wu K., Guo Y., Tan W., Zhang H., Zhang X., Geng G., Pan T., Luo H. (2015). A SnoRNA-derived piRNA interacts with human interleukin-4 pre-mRNA and induces its decay in nuclear exosomes. Nucleic Acids Res..

[66] Ono M., Yamada K., Avolio F., Scott M.S., van Koningsbruggen S., Barton G.J., Lamond A.I. (2010). Analysis of human small nucleolar RNAs (snoRNA) and the development of snoRNA modulator of gene expression vectors. Mol. Biol. Cell.

[67] Mei Y.P., Liao J.P., Shen J., Yu L., Liu B.L., Liu L., Li R.Y., Ji L., Dorsey S.G., Jiang Z.R. (2012). Small nucleolar RNA 42 acts as an oncogene in lung tumorigenesis. Oncogene.

[68] Nogueira Jorge N.A., Wajnberg G., Ferreira C.G., de Sa Carvalho B., Passetti F. (2017). snoRNA and piRNA expression levels modified by tobacco use in women with lung adenocarcinoma. PLoS ONE.

[69] Zheng D., Zhang J., Ni J., Luo J., Wang J., Tang L., Zhang L., Wang L., Xu J., Su B. (2015). Small nucleolar RNA 78 promotes the tumorigenesis in non-small cell lung cancer. J. Exp. Clin. Cancer Res. Cr.

[70] Mannoor K., Shen J., Liao J., Liu Z., Jiang F. (2014). Small nucleolar RNA signatures of lung tumor-initiating cells. Mol. Cancer.

[71] Gong J., Li Y., Liu C.J., Xiang Y., Li C., Ye Y., Zhang Z., Hawke D.H., Park P.K., Diao L. (2017). A Pan-cancer Analysis of the Expression and Clinical Relevance of Small Nucleolar RNAs in Human Cancer. Cell Rep..

[72] Gao L., Ma J., Mannoor K., Guarnera M.A., Shetty A., Zhan M., Xing L., Stass S.A., Jiang F. (2015). Genome-wide small nucleolar RNA expression analysis of lung cancer by next-generation deep sequencing. Int. J. Cancer.

[73] Su Y., Guarnera M.A., Fang H., Jiang F. (2016). Small non-coding RNA biomarkers in sputum for lung cancer diagnosis. Mol. Cancer.

[74] Han Y.N., Li Y., Xia S.Q., Zhang Y.Y., Zheng J.H., Li W. (2017). PIWI Proteins and PIWI-Interacting RNA: Emerging Roles in Cancer. Cell. Physiol. Biochem. Int. J. Exp. Cell. Physiol. Biochem. Pharmacol..

[75] Dorner S., Eulalio A., Huntzinger E., Izaurralde E. (2007). Delving into the diversity of silencing pathways. Symp. Micrornas Sirnas: Biol. Funct. Mech..

[76] Luo S., Lu J. (2017). Silencing of Transposable Elements by piRNAs in Drosophila: An Evolutionary Perspective. Genom. Proteom. Bioinformat..

[77] Siomi M.C., Sato K., Pezic D., Aravin A.A. (2011). PIWI-interacting small RNAs: The vanguard of genome defence. Nat. Rev. Mol. Cell Biol..

[78] Reeves M.E., Firek M., Jliedi A., Amaar Y.G. (2017). Identification and characterization of RASSF1C piRNA target genes in lung cancer cells. Oncotarget.

[79] Mei Y., Wang Y., Kumari P., Shetty A.C., Clark D., Gable T., MacKerell A.D., Ma M.Z., Weber D.J., Yang A.J. (2015). A piRNA-like small RNA interacts with and modulates p-ERM proteins in human somatic cells. Nat. Commun..

[80] Fehon R.G., McClatchey A.I., Bretscher A. (2010). Organizing the cell cortex: The role of ERM proteins. Nat. Rev. Mol. Cell Biol..

[81] Zhang S.J., Yao J., Shen B.Z., Li G.B., Kong S.S., Bi D.D., Pan S.H., Cheng B.L. (2018). Role of piwi-interacting RNA-651 in the carcinogenesis of non-small cell lung cancer. Oncol. Lett..

[82] Li C., Qin F., Hu F., Xu H., Sun G., Han G., Wang T., Guo M. (2018). Characterization and selective incorporation of small non-coding RNAs in non-small cell lung cancer extracellular vesicles. Cell Biosci..

[83] Liao J., Yu L., Mei Y., Guarnera M., Shen J., Li R., Liu Z., Jiang F. (2010). Small nucleolar RNA signatures as biomarkers for non-small-cell lung cancer. Mol. Cancer.

[84] Balbin O.A., Malik R., Dhanasekaran S.M., Prensner J.R., Cao X., Wu Y.M., Robinson D., Wang R., Chen G., Beer D.G. (2015). The landscape of antisense gene expression in human cancers. Genome Res..

[85] Yuan X.S., Cao L.X., Hu Y.J., Bao F.C., Wang Z.T., Cao J.L., Yuan P., Lv W., Hu J. (2017). Clinical, cellular, and bioinformatic analyses reveal involvement of WRAP53 overexpression in carcinogenesis of lung adenocarcinoma. Tumour Biol. J. Int. Soc. Oncodevelopmental Biol. Med..

[86] Shi R., Jiao Z., Yu A., Wang T. (2019). Long noncoding antisense RNA FAM83A-AS1 promotes lung cancer cell progression by increasing FAM83A. J. Cell. Biochem..

[87] He J., Wu K., Guo C., Zhou J.K., Pu W., Deng Y., Zuo Y., Zhao Y., Liu L., Wei Y.Q. (2018). Long non-coding RNA AFAP1-AS1 plays an oncogenic role in promoting cell migration in non-small cell lung cancer. Cell. Mol. Life Sci. Cmls.

[88] Zhang H., Xiong Y., Xia R., Wei C., Shi X., Nie F. (2017). The pseudogene-derived long noncoding RNA SFTA1P is down-regulated and suppresses cell migration and invasion in lung adenocarcinoma. Tumour Biol. J. Int. Soc. Oncodevelopmental Biol. Med..

[89] Sun M., Nie F.Q., Zang C., Wang Y., Hou J., Wei C., Li W., He X., Lu K.H. (2017). The Pseudogene DUXAP8 Promotes Non-small-cell Lung Cancer Cell Proliferation and Invasion by Epigenetically Silencing EGR1 and RHOB. Mol. Ther. J. Am. Soc. Gene Ther..

[90] Wei C.C., Nie F.Q., Jiang L.L., Chen Q.N., Chen Z.Y., Chen X., Pan X., Liu Z.L., Lu B.B., Wang Z.X. (2017). The pseudogene DUXAP10 promotes an aggressive phenotype through binding with LSD1 and repressing LATS2 and RRAD in non small cell lung cancer. Oncotarget.

[91] Gao X., Gao X., Li C., Zhang Y., Gao L. (2016). Knockdown of Long Noncoding RNA uc.338 by siRNA Inhibits Cellular Migration and Invasion in Human Lung Cancer Cells. Oncol. Res..

[92] Vannini I., Wise P.M., Challagundla K.B., Plousiou M., Raffini M., Bandini E., Fanini F., Paliaga G., Crawford M., Ferracin M. (2017). Transcribed ultraconserved region 339 promotes carcinogenesis by modulating tumor suppressor microRNAs. Nat. Commun..

[93] Zhou J., Wang C., Gong W., Wu Y., Xue H., Jiang Z., Shi M. (2018). uc.454 Inhibited Growth by Targeting Heat Shock Protein Family A Member 12B in Non-Small-Cell Lung Cancer. Mol. Ther. Nucleic Acids.

[94] Langley A.R., Chambers H., Christov C.P., Krude T. (2010). Ribonucleoprotein Particles Containing Non-Coding Y RNAs, Ro60, La and Nucleolin Are Not Required for Y RNA Function in DNA Replication. PLoS ONE.

[95] Driedonks T.A.P., Nolte-’t Hoen E.N.M. (2019). Circulating Y-RNAs in Extracellular Vesicles and Ribonucleoprotein Complexes; Implications for the Immune System. Front. Immunol..

[96] Gulei D., Irimie A.I., Cojocneanu-Petric R., Schultze J.L., Berindan-Neagoe I. (2018). Exosomes-Small Players, Big Sound. Bioconjugate Chem..

[97] Braicu C., Tomuleasa C., Monroig P., Cucuianu A., Berindan-Neagoe I., Calin G.A. (2015). Exosomes as divine messengers: Are they the Hermes of modern molecular oncology?. Cell Death Differ..

[98] Werner A., Swan D. (2010). What are natural antisense transcripts good for?. Biochem. Soc. Trans..

[99] Sun Y., Li D., Zhang R., Peng S., Zhang G., Yang T., Qian A. (2017). Strategies to identify natural antisense transcripts. Biochimie.

[100] Zinad H.S., Natasya I., Werner A. (2017). Natural Antisense Transcripts at the Interface between Host Genome and Mobile Genetic Elements. Front. Microbiol..

[101] Nishizawa M., Okumura T., Ikeya Y., Kimura T. (2012). Regulation of Inducible Gene Expression by Natural Antisense Transcripts. Front. Biosci..

[102] Wanowska E., Kubiak M.R., Rosikiewicz W., Makałowska I., Szcześniak M.W. (2018). Natural antisense transcripts in diseases: From modes of action to targeted therapies. Wiley Interdiscip. Rev. RNA.

[103] Kim D.S., Lee W.K., Park J.Y. (2018). Promoter methylation of Wrap53alpha, an antisense transcript of p53, is associated with the poor prognosis of patients with non-small cell lung cancer. Oncol. Lett..

[104] Tutar Y. (2012). Pseudogenes. Comp. Funct. Genom..

[105] Pink R.C., Wicks K., Caley D.P., Punch E.K., Jacobs L., Carter D.R.F. (2011). Pseudogenes: Pseudo-functional or key regulators in health and disease?. RNA.

[106] Cooke S.L., Shlien A., Marshall J., Pipinikas C.P., Martincorena I., Tubio J.M., Li Y., Menzies A., Mudie L., Ramakrishna M. (2014). Processed pseudogenes acquired somatically during cancer development. Nat. Commun..

[107] Shang J., Wang Z., Chen W., Yang Z., Zheng L., Wang S., Li S. (2019). Pseudogene CHIAP2 inhibits proliferation and invasion of lung adenocarcinoma cells by means of the WNT pathway. J. Cell. Physiol..

[108] Xu T., Li D., He Y., Zhang F., Qiao M., Chen Y. (2018). The expression level of CSDAP1 in lung cancer and its clinical significance. Oncol. Lett..

[109] Peng J.C., Shen J., Ran Z.H. (2013). Transcribed ultraconserved region in human cancers. RNA Biol..

[110] Leao R., Apolonio J.D., Lee D., Figueiredo A., Tabori U., Castelo-Branco P. (2018). Mechanisms of human telomerase reverse transcriptase (hTERT) regulation: Clinical impacts in cancer. J. Biomed. Sci..

[111] Webb C.J., Zakian V.A. (2016). Telomerase RNA is more than a DNA template. RNA Biol..

[112] Eldholm V., Haugen A., Zienolddiny S. (2014). CTCF mediates the TERT enhancer-promoter interactions in lung cancer cells: Identification of a novel enhancer region involved in the regulation of TERT gene. Int. J. Cancer.

[113] Ye G., Tan N., Meng C., Li J., Jing L., Yan M., Jin T., Chen F. (2017). Genetic variations in TERC and TERT genes are associated with lung cancer risk in a Chinese Han population. Oncotarget.

[114] Li X., Xu X., Fang J., Wang L., Mu Y., Zhang P., Yao Z., Ma Z., Liu Z. (2016). Rs2853677 modulates Snail1 binding to the TERT enhancer and affects lung adenocarcinoma susceptibility. Oncotarget.

[115] Arinaga M., Shimizu S., Gotoh K., Haruki N., Takahashi T., Takahashi T., Mitsudomi T. (2000). Expression of human telomerase subunit genes in primary lung cancer and its clinical significance. Ann. Thorac. Surg..

